# Reconfigurable Analog Computing Metasurfaces Based on Thin Film Lithium Niobate

**DOI:** 10.1002/nap2.70131

**Published:** 2026-05-09

**Authors:** Huan Chen, Xinghao Duan, Mingbo Zhang, Xin Chen, Junli Wang

**Affiliations:** ^1^ School of Physics Xidian University Xi'an China; ^2^ Guangzhou Institute of Technology Xidian University Guangzhou China

**Keywords:** analog computing, reconfigurable metasurfaces, spatial edge detection, thin film lithium niobate

## Abstract

Analog computing metasurface offers significant advantages, including ultrafast computation, low power consumption, and parallel processing of data, thereby presenting a promising pathway toward the realization of a compact and integrated ultrafast all‐optical information processing system. However, existing designs can only toggle between computing and noncomputing modes, falling short of achieving truly reconfigurable analog computing function. In this work, a reconfigurable transmission‐type analog computing metasurface based on the electro‐optic material thin film lithium niobate is demonstrated in the telecommunication band, aiming to flexibly control the operation functions. Through the dynamic regulation of the external voltages applied to individual structure units, the device transitions its function from first‐order differentiation to either second‐order differentiation or integration operations. The results indicate that the spatial transfer functions can be precisely engineered to meet the requirements for ideal differentiation and integration operations. This methodology facilitates high‐precision spatial signal processing, as validated by inspecting Gaussian signals. Furthermore, the theoretical evaluation based on rectangular signal and complex letter pattern inputs establishes the spatial edge detection resolution of approximately 10 μm for the second‐order differentiation operation. The reconfigurable lithium niobate metasurface signal processor demonstrates considerable potential for applications in fields such as remote sensing and high‐speed image processing.

## Graphical abstract

A transmissive reconfigurable analog computing metasurface based on a simple TFLN structure is demonstrated. The proposed metasurface is truly flexible and capable of switching between different computing functions, such as first‐order differentiation, second‐order differentiation, and integration. Simulations verify its excellent image edge detection performance with a spatial resolution of approximately 10 μm.

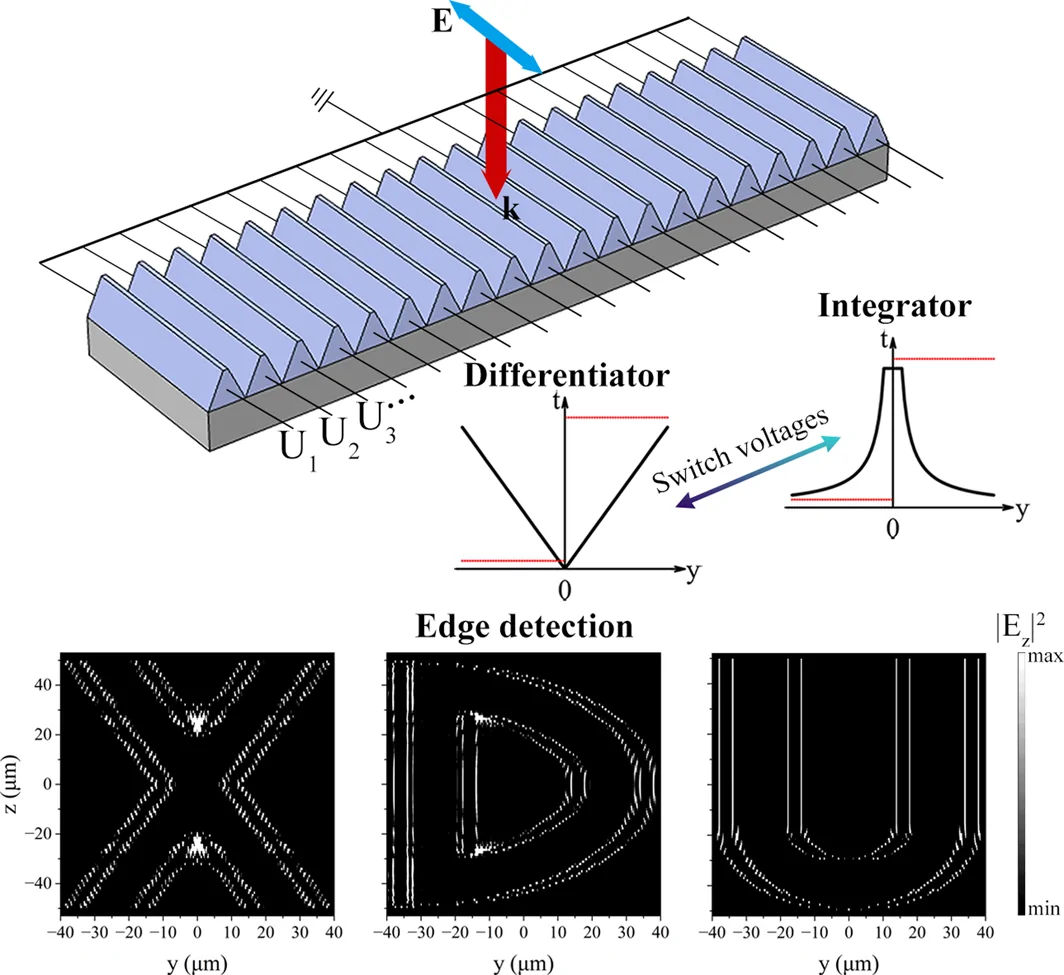

## 1 Introduction

Analog information processing offers faster computation, lower power consumption, higher data fidelity, and parallel data handling by eliminating analog‐to‐digital conversion. Theoretically, these features can address the limitations of digital techniques. However, existing optical transistors and digital logic circuits still face substantial challenges [1]. Conventional analog optical processors inherently suffer from fundamental drawbacks, such as bulky device dimensions and the inability to exceed the diffraction limit [2]. The emergence of metasurfaces, artificially engineered from subwavelength units, has enabled precise light manipulation [3], paving the way for next‐generation optical processors. Through their interaction with light, which induces spatially varying optical responses such as phase, amplitude, and polarization, the metasurfaces can exhibit a wide range of exotic functionalities [2, 4, 5, 6, 7, 8]. In 2014, Silva et al. introduced the concept of “analog computing metamaterials” and proposed a metasurface model capable of performing mathematical operations such as differentiation and integration in the near‐infrared region [9]. Inspired by this pioneering work, researchers have subsequently developed a range of practical analog optical computing metasurfaces [10, 11, 12]. Early analog optical computing metasurfaces were primarily reflection‐type and limited to executing fixed computing functions.

In recent years, research enthusiasm for optical analog computing metasurfaces has surged, leading to the development of various transmission‐type configurations [13, 14, 15, 16, 17, 18, 19]. However, most existing transmission‐type analog computing metasurfaces lack tunable computing function. To address this, researchers have actively pursued multifunctional analog computing metasurfaces to expand the scope of optical information processing [20, 21, 22, 23, 24, 25, 26]. Nevertheless, most tunable transmission‐type metasurfaces rely on the Green's function method to achieve analog computation, rather than modulating the phase and amplitude of light precisely at each structure unit as employed in the 4‐F system approach [9]. Therefore, these designs often require integration with additional components to switch functions or can only toggle between computing and noncomputing modes [20, 24, 25], falling short of achieving truly flexible and reconfigurable analog computing function. This limitation constrains their potential for complex, large‐scale data processing.

Electro‐optic (EO) materials based on the Pockels effect allow the continuous tuning of optical properties via externally applied electric fields. These materials exhibit inherent modulation speeds reaching several hundred gigahertz, exceeding those of thermal and mechanical modulation methods [27]. Among Pockels effect EO materials, thin film lithium niobate (TFLN) is the most widely used for electrically tunable optical metasurfaces, owing to their high refractive index, large EO coefficient, excellent thermal stability, broad optical transparency, and advances in high‐quality TFLN fabrication [27]. To the best of our knowledge, reconfigurable analog computing metasurfaces based on TFLN have not been reported. In this work, we design a novel resonant optical metasurface structure leveraging the tunable optical properties of TFLN. By optimizing its structure parameters, a transmission‐type device operating in the telecommunication band is realized, capable of simultaneous phase and amplitude modulation of transmitted light. Using this tunable TFLN metasurface, we design a first‐order differentiation metasurface by precisely controlling phase and amplitude modulation via applying external voltages. Through the dynamic regulation of the external voltages imposed on the structure units in the metasurface, the device transitions its function from first‐order differentiation to either second‐order differentiation or integration operations. Ultimately, a reconfigurable analog computing TFLN metasurface capable of optical image edge detection is developed.

## 2 Results and Discussion

Figure 1 illustrates the working principle of the reconfigurable analog computing metasurface based on the TFLN. The metasurface consists of TFLN structures with isosceles trapezoidal cross‐sections periodically arranged along the *y*‐ and *z*‐axes and a silica substrate. Each TFLN structure is derived from *x*‐cut TFLN, with an external voltage U_bias_ applied along the *z*‐axis to exploit the largest EO coefficient *γ*_33_. A *z*‐polarized plane wave is normally incident along the −*x*‐axis onto the metasurface. By applying appropriate external voltages to the TFLN structures, the transmission coefficient distribution of the metasurface along the *y*‐axis can be engineered to satisfy the conditions for a differentiator. Adjusting these voltages enables the transmission coefficient distribution to meet the requirements for an integrator. Therefore, switching between different mathematical operations can be achieved simply by tuning the applied voltages, without the need to re‐fabricate the metasurface. The inset within the dashed box in Figure 1 depicts a schematic of the metasurface structure unit. The length (L) of the TFLN structure is set equal to the period (P) of the structure unit, which facilitates subsequent fabrication, testing, and voltage application. In other words, subsequent samples can be fabricated along the *z*‐axis for multiple periods, and the applied voltage only needs to be increased proportionally to the number of periods. The cross‐section of the TFLN structure is designed as an isosceles trapezoid with an inclination angle *θ* = 65°, which is compatible with the existing TFLN etching technologies.

**FIGURE 1:**
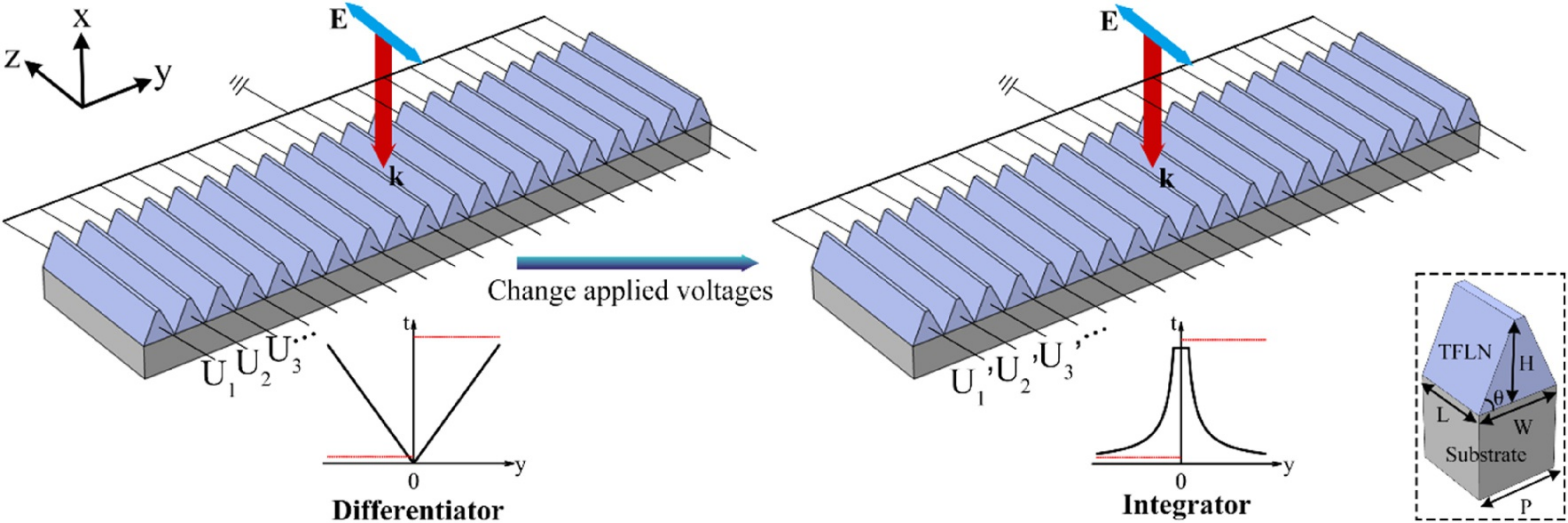
Schematic diagram of a TFLN reconfigurable analog computing metasurface. A *z*‐polarized plane wave is normally incident along the −*x*‐axis onto the TFLN metasurface. When a set of external voltages (U_1_, U_2_, U_3_…) is applied along the *z*‐axis to each structure unit, the metasurface performs a first‐order differentiation operation. When the external voltages applied to each unit are replaced with another set (U_1_′, U_2_′, U_3_′…), the metasurface executes an integration operation. The inset in the black dashed box shows a metasurface structure unit composed of an *x*‐cut TFLN structure and a substrate.

According to the 4‐F system approach [9], to perform first‐order spatial differentiation on a transmitted light, the amplitude and phase modulation of each structure unit must be precisely designed so that the amplitude and phase distributions of the transmission coefficient (i.e., the transfer function) satisfy the following conditions:

(1) $$t_{\text{diff}}\left(y\right)=\left|\frac{t_{\max}y}{l}\right|,$$

(2) $$\left|∠t_{1}-∠t_{2}\right|=180{}^{\circ},$$

where −*l* ≤ *y* ≤ *l*, and 2*l* denotes the total length of the metasurface along the *y*‐axis. *t*_max_ ≤ 1 represents the maximum achievable amplitude of the transmission coefficient; ∠*t*_1_ indicates the phase of the transmission coefficient on the side −*l* ≤ *y* < 0, and ∠*t*_2_ indicates the phase on the side 0 < *y* ≤ *l*. To enable the metasurface to perform second‐order spatial differentiation, the amplitude and phase distributions of the transmission coefficient must satisfy:

(3) $$t_{\text{diff}}\left(y\right)=\frac{t_{\max}y^{2}}{l^{2}},$$

(4) $$∠t_{\text{diff}}=\text{Constant},$$

Equation (4) indicates that the phase of the transmission coefficient remains constant across the entire second‐order differentiation metasurface. For integration, the phase and amplitude distributions must satisfy Equation (2) and

(5) $$t_{\mathrm{int}}\left(y\right)=\left\{\begin{array}{l} t_{\max},\left|y\right|<d\\ \left|\frac{t_{\max}d}{y}\right|,\left|y\right|\geq d \end{array}\right.,$$

here, *2d* (<< *l*) denotes the width of the central region of the metasurface, where the transmission amplitude remains constant at *t*_max_. Therefore, to realize first‐order differentiation, second‐order differentiation, or integration operations, the metasurface structure must be capable of modulating both the amplitude and phase of the transmitted light.

To this end, we design a TFLN metasurface using the commercial simulation software COMSOL Multiphysics. The refractive index of *x*‐cut TFLN is defined as $n=n_{e}-\frac{1}{2}n_{e}^{3}\gamma_{33}e_{z}$, where *n*_*e*_ denotes the extraordinary refractive index of TFLN in the absence of an external electric field and is the interpolated value from the literature [28] (see Supporting Information S1: Figure S1). The EO coefficient *γ*_33_ is set to 30.9 pm/V [29], and *e*_*z*_ represents the electrostatic field generated in the *z*‐axis under an applied voltage. The value of *e*_*z*_ is obtained during the simulation rather than being preset, so the refractive index of TFLN is not entirely preset but varies with changes in the electrostatic field. The substrate is defined as a quartz glass with a refractive index of 1.5. The boundaries of the structure unit along the *y*‐ and *z*‐axes are governed by Floquet periodic conditions, whereas the *x*‐axis is equipped with incident and exit ports, incorporating perfect matched layers at the ports. A *z*‐polarized plane wave is incident normally along the −*x*‐axis onto the metasurface structure unit. Through comprehensive parameter sweeps, we select geometric parameters that enable the metasurface to exhibit resonance near the telecommunication wavelength of 1.55 μm. The optimized structure parameters are *L* = *P* = *W* = 0.96 μm.

In addition, the transmission coefficients of the metasurface are calculated for different structure heights H over the telecommunication band, as shown in Figure 2a,b. These results reveal that the TFLN metasurface exhibits strong localized resonance near 1.55 μm. As H increases, the transmission dip narrows, indicating an increase in the Q‐factor. A high‐Q resonant metasurface is advantageous for enabling a large modulation of the transmitted light and for reducing the required external voltage to achieve the analog computing function. Therefore, H is set to 0.9 μm (as indicated by the white dashed lines in the figures)—the maximum height attainable with the current commercial TFLN technology. These geometric parameters are suitable and practical for fabrication using standard nanofabrication processes.

**FIGURE 2:**
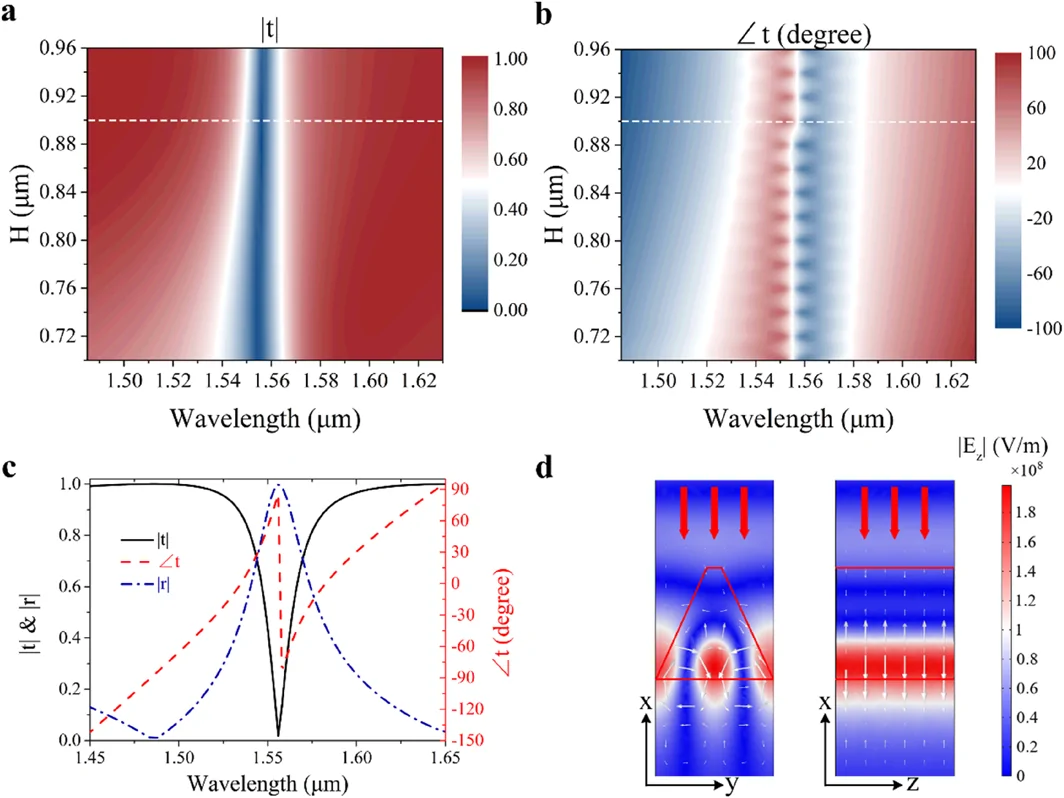
Optimization of TFLN metasurface structure parameters and investigation of the resonance mechanism. (a, b) Transmission spectra of the metasurface under different TFLN structure heights H. The color bars in (a) and (b) represent the amplitude and phase of the transmission coefficient, respectively. The white dashed line indicates *H* = 0.9 μm. (c) Transmission (black solid and red dashed lines) and reflection (blue dash‐dot line) spectra of the TFLN metasurface for *H* = 0.9 μm. (d) Electric field distribution and energy flow (white arrows) at a wavelength of 1.555 μm. The red arrow indicates the incident light, and the red solid line outlines the TFLN structure.

Figure 2c shows the transmission and reflection spectra of the metasurface corresponding to the white dashed line in Figure 2a,b. The results demonstrate that the designed TFLN metasurface allows a wide‐range modulation of transmission amplitude near 1.55 μm and supports a phase modulation range of approximately 180°, meeting the requirements for implementing first‐order differentiation, second‐order differentiation, and integration operations. Furthermore, the transmission dip in the spectrum is asymmetric (also evident in the transmission spectra of Figure 2a), with a minimum transmission coefficient amplitude close to zero. And the position of the dip (i.e., the resonant wavelength) is highly sensitive to changes in structure parameters (see Supporting Information S1: Figure S2). All of these indicate that the resonance is a Fano‐type resonance [30]. To further elucidate the modulation mechanism of the TFLN metasurface, we analyze its field distribution at the resonance wavelength of 1.555 μm, as shown in Figure 2d. The electric field and Poynting vector are primarily concentrated within the TFLN structure and at the TFLN–substrate interface, where the field is strongly localized and enhanced, confirming the occurrence of a strong resonance. Since all materials in the metasurface are defined as lossless, the sharp drop in transmission amplitude at resonance can be attributed to strong reflection (blue dash‐dot line in Figure 2c).

The modulation range of transmission amplitude and phase induced by the Fano resonance satisfies the design requirements for implementing first‐order differentiation, second‐order differentiation, or integration metasurfaces. Furthermore, the sensitivity of Fano resonance to refractive index variations enables the realization of reconfigurable analog computing metasurfaces. By applying an external voltage U_bias_ along the *z*‐axis—grounding the trapezoidal cross‐section on the +*z* side and applying potential U_bias_ to the −*z* side—a static electric field is generated that modulates the refractive index of TFLN, thereby altering the resonance characteristics of the metasurface. The relationship between the applied external voltage U_bias_ and the metasurface transmission spectrum is shown in Figure 3a,b. As observed, increasing U_bias_ results in a pronounced blue shift of the resonant wavelength. Near resonance, a phase shift of approximately 180° occurs in the transmitted light.

**FIGURE 3:**
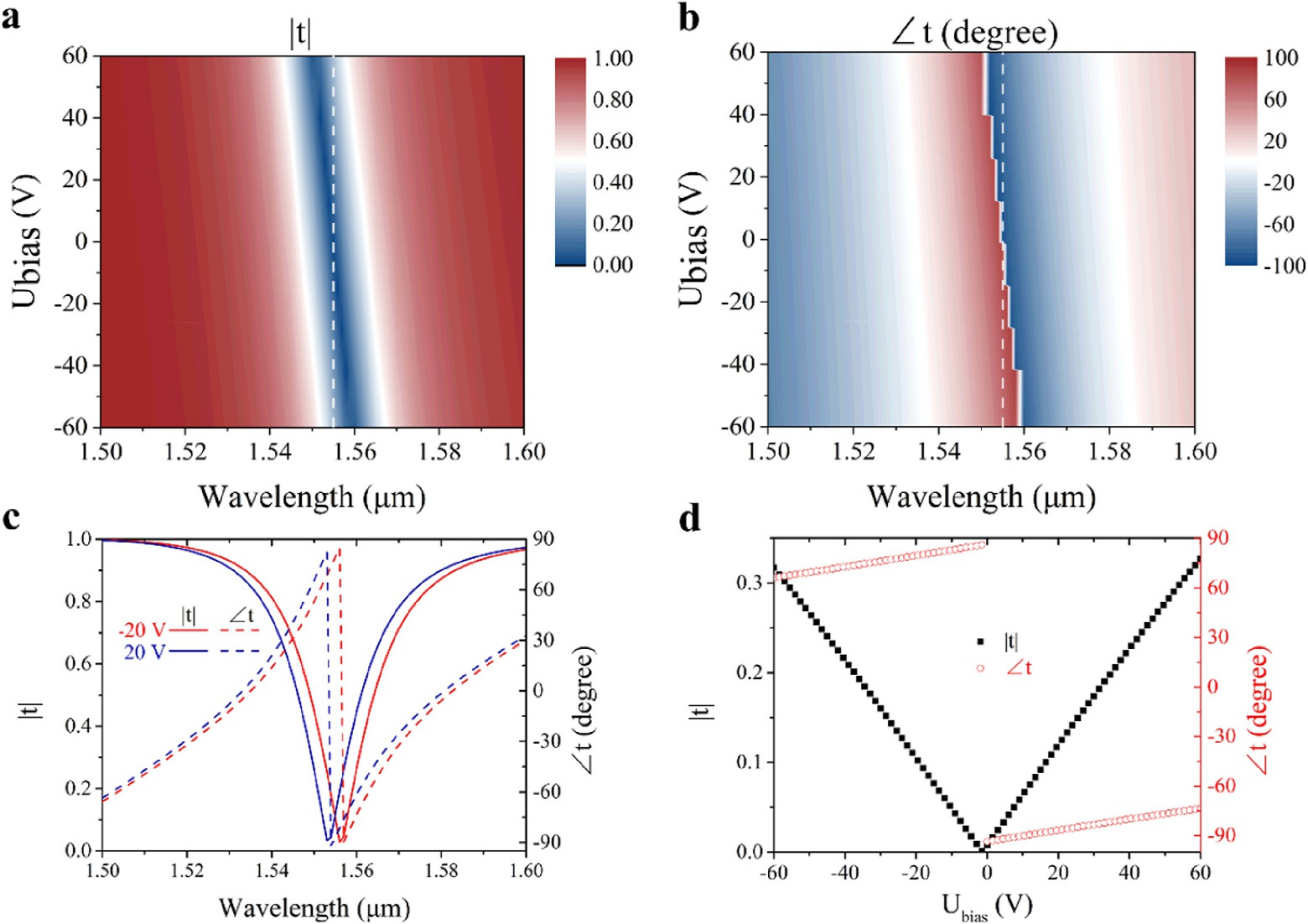
Investigation of the electrically tunable characteristics of the TFLN metasurface. (a, b) Transmission spectra of the metasurface under various external voltages U_bias_. The color bars in (a) and (b) represent the amplitude and phase of the transmission coefficient, respectively. The white dashed line corresponds to the wavelength of 1.555 μm. (c) Transmission spectra at U_bias_ = +20 V (blue lines) and −20 V (red lines). Solid lines indicate transmission amplitude, and dashed lines indicate the transmission phase. (d) Relationship between the transmission coefficient and U_bias_ at a fixed incident wavelength of 1.555 μm. Black solid squares denote amplitude; red open circles denote phase.

Figure 3c presents the transmission spectra of the metasurface under U_bias_ = ± 20 V, further confirming the significant influence of the applied voltage on the resonance characteristics. This demonstrates that, at a fixed telecommunication wavelength, large simultaneous modulation of transmission amplitude and phase can be achieved by tuning U_bias_. Figure 3d displays the relationship between the transmission coefficient and the applied voltage at the resonance wavelength of 1.555 μm (corresponding to the white dashed line in Figure 3a,b). As the voltage varies from −60 V to +60 V, the phase of the transmission coefficient changes by approximately 180°, and the amplitude varies from 0 to 0.32. Considering the risk of domain inversion [31] in TFLN under high voltage which could alter both the refractive index and *γ*_33_, the maximum transmission amplitude *t*_max_ is conservatively set to 0.2. Under this constraint, the practical tuning range of the external voltage is approximately −35 V to +35 V.

Using the relationship between the applied voltage and the transmission coefficient shown in Figure 3d, and setting *t*_max_ to 0.2, we design a metasurface consisting of 80 identical structure units along the *y*‐axis. Appropriate external voltages are applied to the TFLN in each structure unit (see Supporting Information S1: Table S1). A *z*‐polarized plane wave is normally incident along the −*x*‐axis onto the metasurface, and the simulated distribution of the transmitted electric field E_z_ is shown in Figure 4a. The green dashed box marks the TFLN metasurface, with E_z_ illustrated below. It is observed that, with the metasurface center defined as *y* = 0, the transmitted field exhibits opposite phases on the *y* < 0 and *y* > 0 sides, and the closer to the center of the metasurface, the weaker the transmitted electric field becomes. This confirms that the transmission coefficient distribution satisfies Equations (1) and (2), verifying that the TFLN metasurface operates as a first‐order differentiator. The line profiles of E_z_ at the output port along the *y*‐axis further validate the first‐order differentiation function of the TFLN metasurface. Aside from field attenuation near the metasurface boundaries—attributable to interference caused by the periodic boundary conditions (the transmission amplitudes at both boundaries are designed to be equal, but their phases are opposite)—the simulation results are largely consistent with theoretical predictions. To further confirm its computing performance, we replace the input plane wave with a Gaussian beam signal exp[−(*y*/w_1_)^2^] (w_1_ = 15 μm is the beam waist), as shown in Figure 4d. The amplitude distribution of the output signal along the *y*‐axis is displayed in Figure 4e. Apart from a slight displacement of the curve in the *y*‐axis (Δ*y* ≈ −10 μm, attributable to minor deviations between the actual transmission coefficient distribution and the theoretical profile [32]), the simulation result is in good agreement with the theoretical prediction. This provides additional evidence that the designed TFLN metasurface performs first‐order differentiation as intended.

**FIGURE 4:**
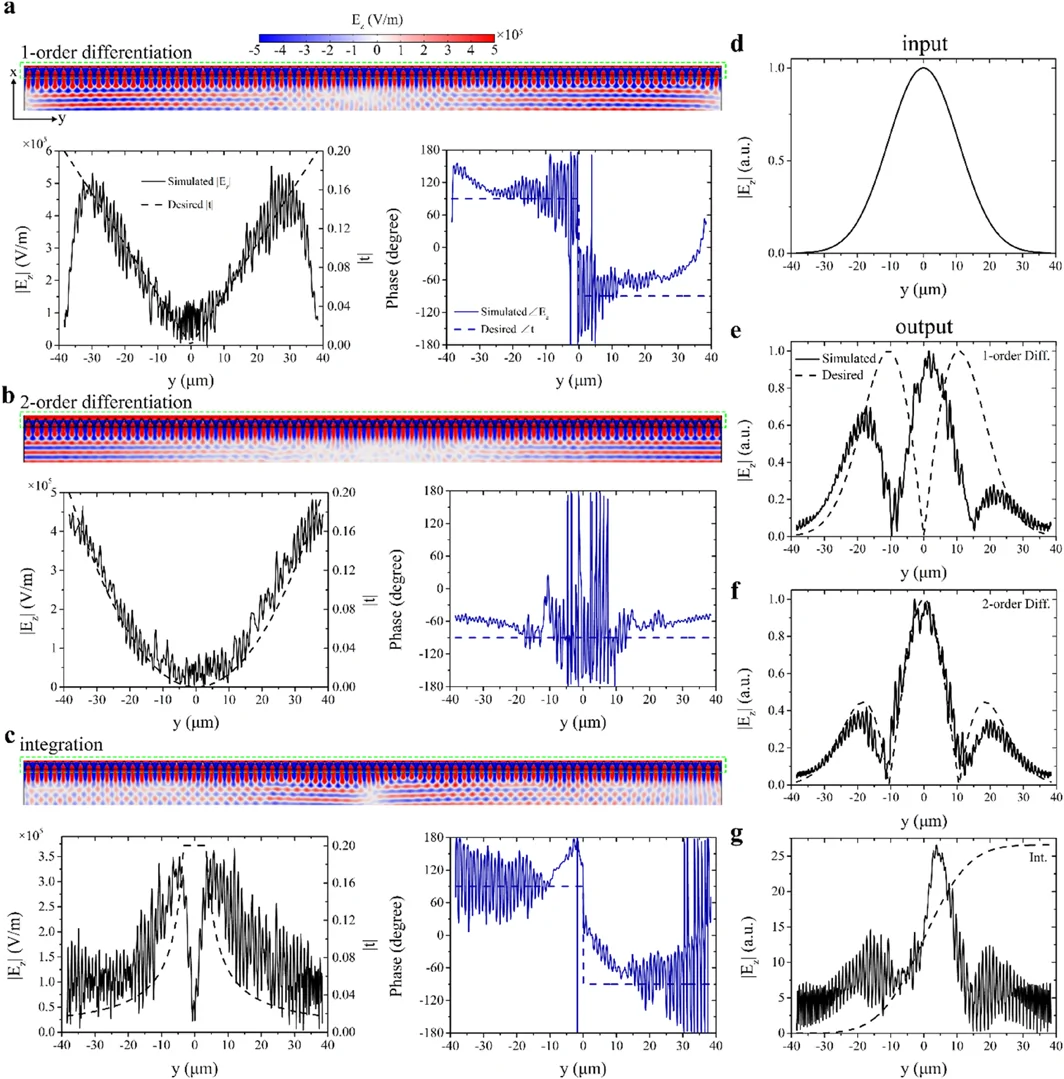
Simulation of the first‐order differentiation, second‐order differentiation, and integration TFLN metasurfaces. (a–c) Transmitted electric field E_z_ distributions in the *y*–*x* plane (*z* = 0) and line profiles of the amplitude (black solid lines) and phase (blue solid lines) of the transmitted electric field E_z_ distributions for the first‐order differentiation, second‐order differentiation, and integration TFLN metasurfaces, respectively. Black and blue dashed lines represent theoretical predictions. (d) Input Gaussian beam signal exp[−(*y*/w_1_)^2^] (w_1_ = 15 μm). (e–g) Amplitude distributions of the output signals along the *y*‐axis after passing through the first‐order differentiation, second‐order differentiation, and integration TFLN metasurfaces, respectively. Black solid line: simulation result; black dashed line: theoretical prediction.

To reconfigure the computing function of the TFLN metasurface to perform second‐order differentiation, we again utilize the relationship between the applied voltage and the transmission coefficient shown in Figure 3d. *t*_max_ is set to 0.2, and the transmission phase ∠*t*_*diff*_ is fixed at −90°. Appropriate external voltages are applied to the TFLN structure units (see Supporting Information S1: Table S2). A *z*‐polarized plane wave is normally incident along the −*x‐*axis onto the metasurface, and the simulated E_z_ distribution is shown in Figure 4b. As indicated, the transmission phases on the *y* < 0 and *y* > 0 sides are nearly identical, whereas the transmission amplitude decreases toward the center. This confirms that the metasurface transmission coefficient satisfies Equations (3) and (4), validating the second‐order differentiation behavior of the TFLN metasurface. The line profiles of E_z_ also agree with theoretical predictions, further confirming the second‐order differentiation function of the TFLN metasurface. To demonstrate its practical computing capability, the incident plane wave is replaced with the Gaussian beam signal exp[−(*y*/w_1_)^2^] (w_1_ = 15 μm). As shown in Figure 4f, the amplitude distribution of the output signal closely matches the theoretical prediction, providing further evidence that the designed TFLN metasurface realizes second‐order differentiation.

The relationship between the applied voltage and the transmission coefficient shown in Figure 3d is employed again to reconfigure the TFLN metasurface to perform integration. Set *t*_max_ = 0.2 and *d* = 4*P*, and apply appropriate external voltages to the TFLN structure units (see Supporting Information S1: Table S3). The simulated E_z_ distribution is shown in Figure 4c. As illustrated, the transmitted E_z_ on the *y* < 0 and *y* > 0 sides exhibits opposite phases, whereas the amplitude increases toward the center, except in the immediate vicinity of *y* = 0 where interference due to opposite phases weakens the field. This indicates that the transmission coefficient distribution satisfies Equations (2) and (5), confirming that the TFLN metasurface functions as an integrator. Except for the localized reduction in amplitude near *y* = 0 caused by interference, the line profiles of E_z_ are in close agreement with theoretical predictions, further validating the integration function of the TFLN metasurface. Similarly, to demonstrate the practical computing capability of the metasurface integrator, we replace the plane wave input with the Gaussian beam signal exp[−(*y*/w_1_)^2^] (w_1_ = 15 μm). The amplitude distribution of the output signal is presented in Figure 4g. Due to interference near *y* = 0, the actual transmission coefficient distribution deviates somewhat from the theoretical profile, resulting in noticeable discrepancies between the simulation result and the theoretical prediction. Nevertheless, these results qualitatively reflect the expected integration behavior. For this analog computing metasurface, there is near‐field coupling between structure units, but the results show that the transmission coefficient distributions (i.e., transfer functions) of the metasurface still match well with the desired ones. This indicates that the near‐field coupling between structure units has a small impact on the final transfer function. Moreover, electrostatic field coupling leads to an inhomogeneous field distribution in the TFLN structure, which in turn results in an inhomogeneous refractive index distribution (see Supporting Information S1: Figure S3). Nevertheless, simulations show that this inhomogeneity does not compromise the metasurface's analog computing functions, particularly its first‐ and second‐order differentiation capabilities.

Second‐order differentiation metasurfaces are expected to exhibit edge detection capability [14, 19, 21, 24]. To verify this capability of the second‐order differentiation TFLN metasurface, we set the input signal to a rectangular profile $E_{z}=\left\{\begin{array}{l} 1,\left|y\right|\leq w_{2}\\ 0,\left|y\right|>w_{2} \end{array}\right.$, as shown in Figure 5a–c. The widths of the rectangular signals, 2w_2_, are 40 μm, 10 μm, and 6 μm, respectively. Figure 5d–f shows the corresponding transmitted electric field intensity |E_z_|^2^ distributions in the *y–z* plane at the output port after propagation through the second‐order differentiation TFLN metasurface. For clarity, the field intensity distributions in Figure 5a–f are generated by repeating 20 times along the *z*‐axis. Bright lines appear near the edges of the rectangular signals, indicating that the metasurface successfully highlights abrupt intensity transitions, thereby detecting the signal edges. As the width of the input rectangular signal is reduced to 6 μm, the edges become indistinguishable. This observation is further confirmed by the line profiles of the transmitted field intensity |E_z_|^2^ along the *y*‐axis, as shown in Figure 5g–i. Based on these results, the minimum detectable signal width of the proposed second‐order differentiation TFLN metasurface is approximately 10 μm. As demonstrated in the comparison between the input and output signals in Figure 5g,h, the edges of the input signals are in the dark regions between the bright regions after being processed by the metasurface, which is in line with the theoretical prediction. Therefore, the edge information of the input signal can be extracted by analyzing the dark regions between the bright regions in the output image. Simulations performed using only the substrate without the TFLN structures (see Supporting Information S1: Figure S4) further support the edge detection capability of the second‐order differentiation TFLN metasurface. The minimum feature size that the metasurface can resolve in edge detection is larger than the working wavelength. Its resolving power is indeed inferior to that of devices such as metalenses. However, our purpose here is not to demonstrate ultra‐high resolution but to validate its optical signal processing capability. With the necessary signal processing capabilities, it potentially enables parallel, ultrafast processing of large‐scale, high‐volume signals.

**FIGURE 5:**
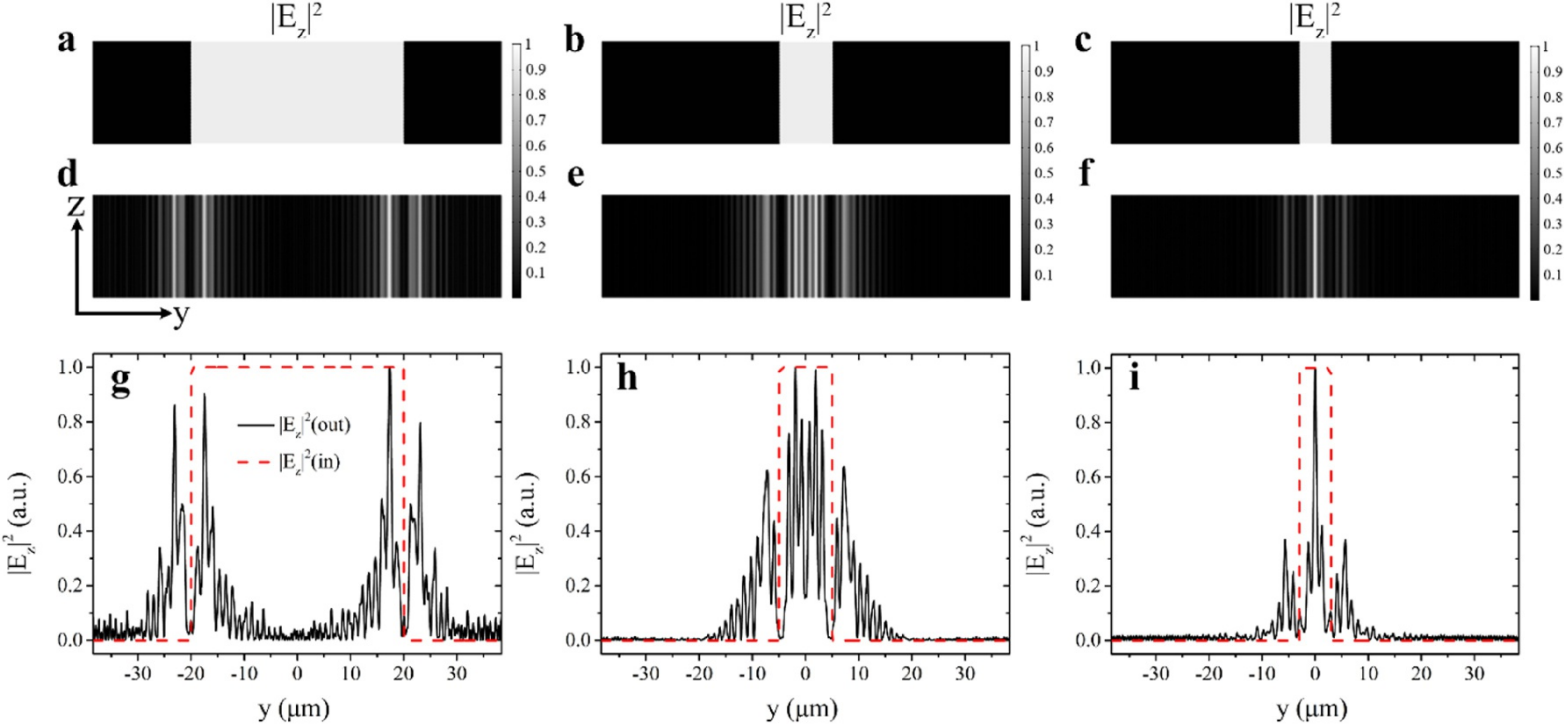
Edge detection capability of the second‐order differentiation TFLN metasurface. (a–c) Electric field intensity |E_z_|^2^ distributions of the input rectangular signals in the *y–z* plane, with signal widths 2w_2_ = 40 μm, 10 μm, and 6 μm. (d–f) Transmitted field intensity |E_z_|^2^ distributions in the *y–z* plane at the output port for the input rectangular signals in (a)–(c). For clearer visualization, the field intensity patterns in (a)–(f) are generated by repeating 20 times along the *z*‐axis. (g–i) Line profiles (black solid lines) along the *y*‐axis of the transmitted electric field intensity |E_z_|^2^ distributions in (d)–(f). Red dashed lines indicate the profiles of the input rectangular signals.

We further demonstrate the robustness and versatility of the edge detection performance for the second‐order differentiation TFLN metasurface by inputting complex optical images of letters X, D, and U, as shown in Figure 6. The input images of the letters X, D, and U represent the abbreviations of our institution, as shown in Figure 6a–c. As the size of the previously designed metasurface is close to that of the letter image, a second‐order differentiation TFLN metasurface composed of 120 structure units is redesigned to avoid noise caused by the interferences at the periodic boundaries. This metasurface follows the same design principles as the previously described second‐order differentiation metasurface. For these 2D images, second‐order differentiation is performed line by line along the *y*‐axis. The resulting data are consequently normalized line by line, as shown in Figure 6d–f. Although two superfluous bright lines are present at the vertical boundaries of the letter D, the overall outcomes are consistent with the second‐order differentiation theory and demonstrate satisfactory edge detection performance. In addition, due to the limited number of lines during the line‐by‐line image processing, the edge detection results exhibit a slightly discrete appearance. Furthermore, to quantitatively evaluate the edge detection performance of the metasurface, the signal‐to‐noise ratio (SNR) of the edge detection results for the letter images is analyzed, as shown in Figure 6g–i. First, the SNR (=10log_10_(*I*_S_/*I*_N_)) for each line in the line‐by‐line processing results—calculated from the average signal intensity *I*_S_ and average noise intensity *I*_N_ per line—is plotted. Subsequently, the average of these SNR values is derived, as indicated by SNR_avg_. These results show that the SNR_avg_ for each letter image is approximately 12 dB, which falls within an acceptable range. Based on both the visual outcomes and the SNR values, it can be concluded that the edge detection performance of the second‐order differentiation TFLN metasurface is reliable.

**FIGURE 6:**
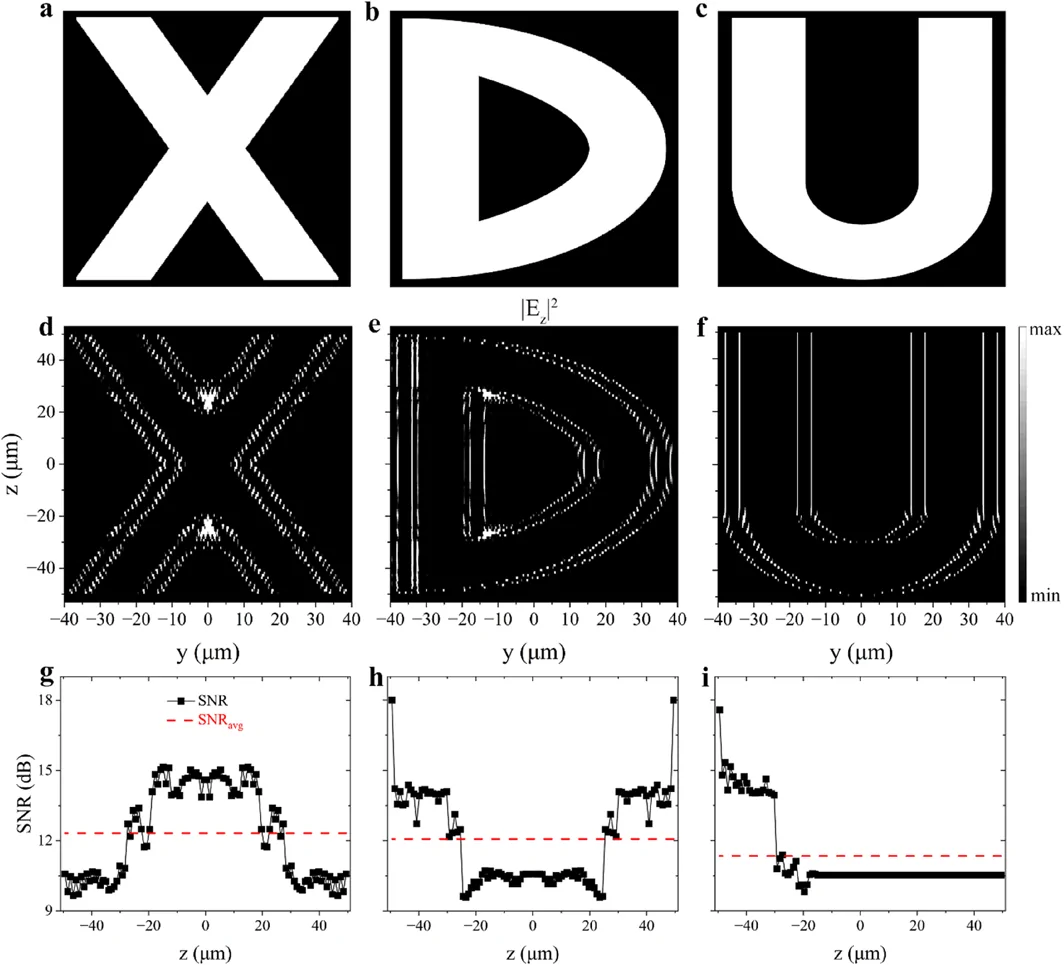
Edge detection performance of the second‐order differentiation TFLN metasurface for complex optical image inputs. (a–c) The input images and (d–f) the corresponding output images for the second‐order differentiation TFLN metasurface. The input images are the letters X, D, and U, respectively. (g–i) The SNR of the edge detection results for the letter images X, D, and U, respectively. The black square lines represent the SNR for each line in the line‐by‐line processing results; the red dashed lines represent the average of these SNR values.

It should be noted that the first‐order differentiation TFLN metasurface presented in this work also exhibits edge detection capability, with performance comparable to that of the second‐order differentiation metasurface (see Supporting Information S1: Figure S5). In addition, because the proposed TFLN metasurfaces are electrically tunable, the operating wavelengths of the differentiator and integrator are also tunable, as demonstrated by the tunable second‐order differentiation TFLN metasurface (see Supporting Information S1: Figure S6). However, due to the potential risk of domain inversion in TFLN under high voltage, excessive external voltage is not applied in this work. Therefore, the tuning range of the operating wavelength for the second‐order differentiation metasurface is relatively limited. Future work may extend this tuning range by further optimizing the metasurface structure parameters, including structure shape and geometry parameters, to develop TFLN analog computing metasurfaces with broader wavelength tunability. The TFLN analog computing metasurface is designed to operate around 1.555 μm, and it loses its original analog computing function under large incident wavelength deviations (see Supporting Information S1: Figure S7). The TFLN metasurface can only perform one‐dimensional (along the *y*‐axis) analog computing. Expanding it to a two‐dimensional situation will definitely increase the difficulty and complexity of the design and pose challenges for subsequent sample fabrication.

Since our metasurface structure is similar to a grating structure, with voltages applied to the two end faces of the trapezoidal cross‐section LN pillar, we need only to deposit electrodes on the end faces of the pillars during actual sample fabrication. The specific electrode and circuit can refer to the design in the literature [33]. Compared to the ubiquitous electronic devices in daily life, the control circuit for this metasurface is relatively simple. Admittedly, although this reconfigurable metasurface is more complex than its fixed‐function counterparts, it provides significantly enhanced function diversity. Furthermore, since no additional components are introduced into the optical path, the computing speed remains unaffected. In this study, a novel reconfigurable analog computing TFLN metasurface is successfully designed through numerical simulations. However, experimental validation remains a crucial next step for assessing its practical performance. The TFLN metasurface is fully compatible with existing manufacturing technologies, as it is designed based on realistic fabrication processes. Nonetheless, the structure parameters of the fabricated metasurface sample may deviate from the design specifications, and the actual electro‐optic effect of TFLN may also differ from theoretical predictions. Therefore, our future research will focus on fabricating samples and conducting iterative comparisons between simulation and experimental results along with corresponding adjustments to verify the simulation results presented in this work.

## 3 Conclusion

In summary, this work demonstrates a reconfigurable analog computing metasurface capable of performing multiple mathematical operations by exploiting the electrically tunable optical properties of TFLN. By adjusting the external voltages applied to structure units, the TFLN metasurface can execute first‐order differentiation, second‐order differentiation, and integration operations. Extensive simulations confirm that the metasurface's transfer functions (i.e., the transmission coefficient distributions) closely match theoretical predictions. When a Gaussian beam serves as the input signal, the simulated first‐order and second‐order differentiation functions of the metasurface are consistent with theoretical expectations, whereas the integration results display more pronounced deviations due to greater disparities between the actual and theoretical transfer functions. The edge detection capability of the metasurface in its second‐order differentiation configuration is validated by inputting rectangular signals and complex letter patterns, establishing a minimum detectable width of approximately 10 μm. The proposed reconfigurable metasurface device offers significant potential for a broad range of applications, including remote sensing and high‐speed image processing. Owing to the excellent characteristics of TFLN—such as integration convenience and compatibility with other photonic platforms—this work offers a promising platform for advancing optical computing and signal processing.

## Author Contributions

**Huan Chen:** conceptualization, methodology, software, data curation, investigation, validation, formal analysis, funding acquisition, visualization, project administration, resources, writing – original draft, writing – review and editing. **Xinghao Duan:** data curation, investigation, validation. **Mingbo Zhang:** data curation, investigation, validation. **Xin Chen:** supervision, funding acquisition, project administration, resources, writing – review and editing. **Junli Wang:** supervision, funding acquisition, project administration, resources, writing – review and editing.

## Funding

The authors acknowledge the funding provided by the Natural Science Foundation of Shaanxi Province (Grants 2025JC‐YBQN‐910, 2023‐JC‐QN‐0014), the National Natural Science Foundation of China (Grant 62275209), the Natural Science Foundation of Guangdong Province (Grant 2023A1515012928), and the Fundamental Research Funds for the Central Universities (Grant ZYTS25126).

## Conflicts of Interest

The authors declare no conflicts of interest.

## Supporting information


Supporting Information S1

## Data Availability

The data that support the findings of this study are available from the corresponding author upon reasonable request.

## References

[1] D. A. B. Miller , “Are Optical Transistors the Logical Next Step?,” Nature Photonics 4, no. 1 (2010): 3–5, 10.1038/nphoton.2009.240.

[2] N. F. Yu , P. Genevet , F. Aieta , et al., “Flat Optics: Controlling Wavefronts With Optical Antenna Metasurfaces,” IEEE Journal of Selected Topics in Quantum Electronics 19, no. 3 (2013): 4700423, 10.1109/JSTQE.2013.2241399.

[3] N. F. Yu , P. Genevet , M. A. Kats , et al., “Light Propagation With Phase Discontinuities: Generalized Laws of Reflection and Refraction,” Science 334, no. 6054 (2011): 333–337, 10.1126/science.1210713. 21885733

[4] S. Sun , Q. He , S. Xiao , Q. Xu , X. Li , and L. Zhou , “Gradient‐Index Meta‐Surfaces as a Bridge Linking Propagating Waves and Surface Waves,” Nature Materials 11, no. 5 (2012): 426–431, 10.1038/nmat3292. 22466746

[5] X. Ni , A. V. Kildishev , and V. M. Shalaev , “Metasurface Holograms for Visible Light,” Nature Communications 4, no. 1 (2013): 2807, 10.1038/ncomms3807.

[6] D. Lin , P. Fan , E. Hasman , and M. L. Brongersma , “Dielectric Gradient Metasurface Optical Elements,” Science 345, no. 6194 (2014): 298–302, 10.1126/science.1253213. 25035488

[7] X. Ni , Z. J. Wong , M. Mrejen , Y. Wang , and X. Zhang , “An Ultrathin Invisibility Skin Cloak for Visible Light,” Science 349, no. 6254 (2015): 1310–1314, 10.1126/science.aac9411. 26383946

[8] M. Khorasaninejad , W. T. Chen , R. C. Devlin , J. Oh , A. Y. Zhu , and F. Capasso , “Metalenses at Visible Wavelengths: Diffraction‐Limited Focusing and Subwavelength Resolution Imaging,” Science 352, no. 6290 (2016): 1190–1194, 10.1126/science.aaf6644. 27257251

[9] A. Silva , F. Monticone , G. Castaldi , V. Galdi , A. Alù , and N. Engheta , “Performing Mathematical Operations With Metamaterials,” Science 343, no. 6167 (2014): 160–163, 10.1126/science.1242818. 24408430

[10] A. Pors , M. G. Nielsen , and S. I. Bozhevolnyi , “Analog Computing Using Reflective Plasmonic Metasurfaces,” Nano Letters 15, no. 1 (2015): 791–797, 10.1021/nl5047297. 25521830

[11] A. Chizari , S. Abdollahramezani , M. V. Jamali , and J. A. Salehi , “Analog Optical Computing Based on a Dielectric Meta‐Reflect Array,” Optics Letters 41, no. 15 (2016): 3451–3454, 10.1364/ol.41.003451. 27472591

[12] H. Chen , D. An , Z. C. Li , and X. P. Zhao , “Performing Differential Operation With a Silver Dendritic Metasurface at Visible Wavelengths,” Optics Express 25, no. 22 (2017): 26417–26426, 10.1364/oe.25.026417. 29092132

[13] H. Kwon , D. Sounas , A. Cordaro , A. Polman , and A. Alù , “Nonlocal Metasurfaces for Optical Signal Processing,” Physical Review Letters 121, no. 17 (2018): 173004, 10.1103/PhysRevLett.121.173004. 30411907

[14] A. Cordaro , H. Kwon , D. Sounas , A. F. Koenderink , A. Alù , and A. Polman , “High‐Index Dielectric Metasurfaces Performing Mathematical Operations,” Nano Letters 19, no. 12 (2019): 8418–8423, 10.1021/acs.nanolett.9b02477. PMC6909238 31675241

[15] D. Pan , L. Wan , M. Ouyang , et al., “Laplace Metasurfaces for Optical Analog Computing Based on Quasi‐Bound States in the Continuum,” Photonics Research 9, no. 9 (2021): 1758, 10.1364/prj.426827.

[16] Z. C. Wang , G. W. Hu , X. W. Wang , et al., “Single‐Layer Spatial Analog Meta‐Processor for Imaging Processing,” Nature Communications 13, no. 1 (2022): 2188, 10.1038/s41467-022-29732-4. PMC9023575 35449139

[17] A. Cordaro , B. Edwards , V. Nikkhah , A. Alo , N. Engheta , and A. Polman , “Solving Integral Equations in Free Space With Inverse‐Designed Ultrathin Optical Metagratings,” Nature Nanotechnology 18, no. 4 (2023): 365–372, 10.1038/s41565-022-01297-9. 36635333

[18] D. Xu , W. Xu , Q. Yang , W. Zhang , S. Wen , and H. Luo , “All‐Optical Object Identification and Three‐Dimensional Reconstruction Based on Optical Computing Metasurface,” Opto‐Electronic Advances 6, no. 12 (2023): 230120, 10.29026/oea.2023.230120.

[19] C. Zhou , Y. Chen , Y. Li , et al., “Laplace Differentiator Based on Metasurface With Toroidal Dipole Resonance,” Advanced Functional Materials 34, no. 19 (2024): 2313777, 10.1002/adfm.202313777.

[20] T. T. Xiao , H. Yang , Q. Yang , et al., “Realization of Tunable Edge‐Enhanced Images Based on Computing Metasurfaces,” Optics Letters 47, no. 4 (2022): 925–928, 10.1364/ol.450988. 35167560

[21] X. Liang , Z. Zhou , Z. Li , et al., “All‐Optical Multiplexed Meta‐Differentiator for Tri‐Mode Surface Morphology Observation,” Advanced Materials 35, no. 29 (2023): 2301505, 10.1002/adma.202301505. 37203240

[22] H. Yang , Y. Chen , Y. Wu , Y. Hu , J. Yang , and J. Wu , “Analog Signal Metasurface Processor Supporting Mathematical Operator Reconfiguration,” Optics Letters 48, no. 21 (2023): 5451, 10.1364/ol.498519. 37910675

[23] Y. Zhou , J. Zhan , Y. Shao , et al., “Reconfigurable Spatiotemporal Optical Signal Processors,” Advanced Optical Materials 11, no. 21 (2023): 2300746, 10.1002/adom.202300746.

[24] M. Cotrufo , S. B. Sulejman , L. Wesemann , et al., “Reconfigurable Image Processing Metasurfaces With Phase‐Change Materials,” Nature Communications 15, no. 1 (2024): 4483, 10.1038/s41467-024-48783-3. PMC11130277 38802353

[25] A. Long , Z. Xie , H. Wu , et al., “Switchable Optical Differentiator Using Fano Resonance Metasurface,” Optics Communications 550 (2024): 129954, 10.1016/j.optcom.2023.129954.

[26] C. Peng , T. Huang , C. Chen , et al., “Switchable Two‐Dimensional AND and Exclusive OR Operation Based on Dual‐Wavelength Metasurfaces,” ACS Nano 18, no. 5 (2024): 4424–4431, 10.1021/acsnano.3c10723. 38276787

[27] F. Ding , C. Meng , and S. I. Bozhevolnyi , “Electrically Tunable Optical Metasurfaces,” Photonics Insights 3, no. 3 (2024): R07, 10.3788/pi.2024.r07.

[28] D. E. Zelmon , D. L. Small , and D. Jundt , “Infrared Corrected Sellmeier Coefficients for Congruently Grown Lithium Niobate and 5 Mol% Magnesium Oxide–Doped Lithium Niobate,” Journal of the Optical Society of America B 14, no. 12 (1997): 3319–3322, 10.1364/josab.14.003319.

[29] G. Chen , N. Li , J. D. Ng , et al., “Advances in Lithium Niobate Photonics: Development Status and Perspectives,” Advanced Photonics 4, no. 3 (2022): 034003, 10.1117/1.AP.4.3.034003.

[30] M. F. Limonov , M. V. Rybin , A. N. Poddubny , and Y. S. Kivshar , “Fano Resonances in Photonics,” Nature Photonics 11, no. 9 (2017): 543–554, 10.1038/nphoton.2017.142.

[31] L. Chang , Y. Li , N. Volet , L. Wang , J. Peters , and J. E. Bowers , “Thin Film Wavelength Converters for Photonic Integrated Circuits,” Optica 3, no. 5 (2016): 531–535, 10.1364/optica.3.000531.

[32] N. V. Golovastikov , L. L. Doskolovich , E. A. Bezus , D. A. Bykov , and V. A. Soifer , “An Optical Differentiator Based on a Three‐Layer Structure With a W‐Shaped Refractive Index Profile,” Journal of Experimental and Theoretical Physics 127, no. 2 (2018): 202–209, 10.1134/s1063776118080174.

[33] G. K. Shirmanesh , R. Sokhoyan , P. C. Wu , and H. A. Atwater , “Electro‐Optically Tunable Multifunctional Metasurfaces,” ACS Nano 14, no. 6 (2020): 6912–6920, 10.1021/acsnano.0c01269. 32352740

