# Supplementary material for: Reconfigurable Analog Computing Metasurfaces Based on Thin Film Lithium Niobate
Source: Nanophotonics. 2026 May 9;15(9):e70131. doi: 10.1002/nap2.70131 (PMC13642559; doi:10.1002/nap2.70131)
Supplement: Supplementary file 1 [file NAP2-15-e70131-s001.pdf]

Supporting Information

Reconfigurable Analog Computing Metasurfaces Based on Thin Film Lithium Niobate

Huan Chen, Xinghao Duan, Mingbo Zhang, Xin Chen*, Junli Wang**

Section S1: Refractive indexes of TFLN

In this work, the extraordinary refractive indexes n_e of TFLN are set to the values reported by Zelmon et al. in the literature[1] (see **Figure S1**), which are measured via the minimum-deviation technique. Furthermore, TFLN is treated as a lossless material, implying that the imaginary part of n_e is zero.

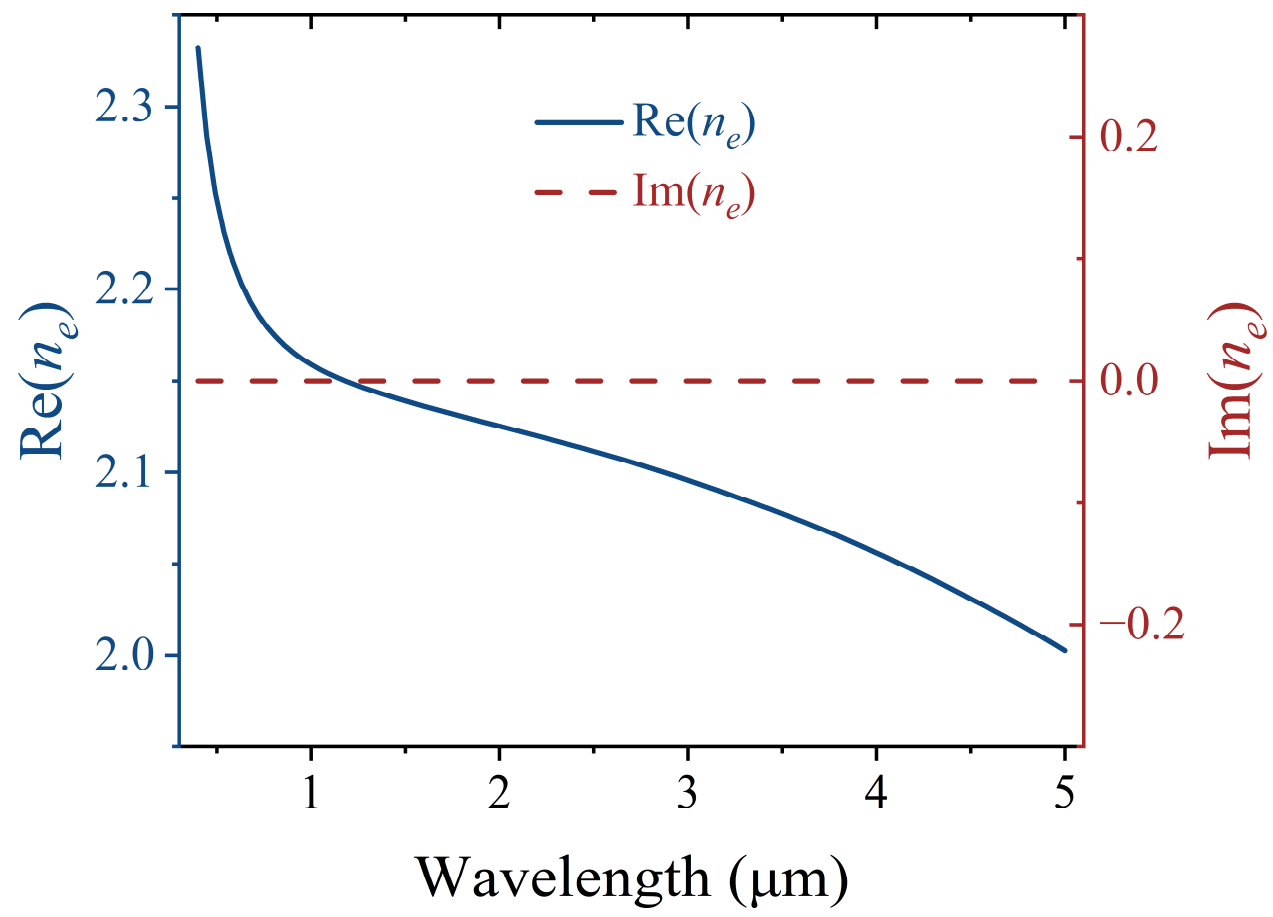

Figure S1. Refractive indexes of TFLN. The blue solid line and red dash line represent the real and imaginary parts of refractive indexes, respectively.

Section S2: Impact of structure parameters on the resonance of TFLN metasurface

The metasurface structure designed in this work exhibits Fano resonance behavior, characterized by an asymmetric transmission dip that is highly sensitive to structure parameters, as shown in **Figure S2**. It can be observed that slight variations in the period P, width W, and tilt angle θ induce significant shifts in the resonant wavelength of the metasurface structure, while the transmission dips remain consistently zero.

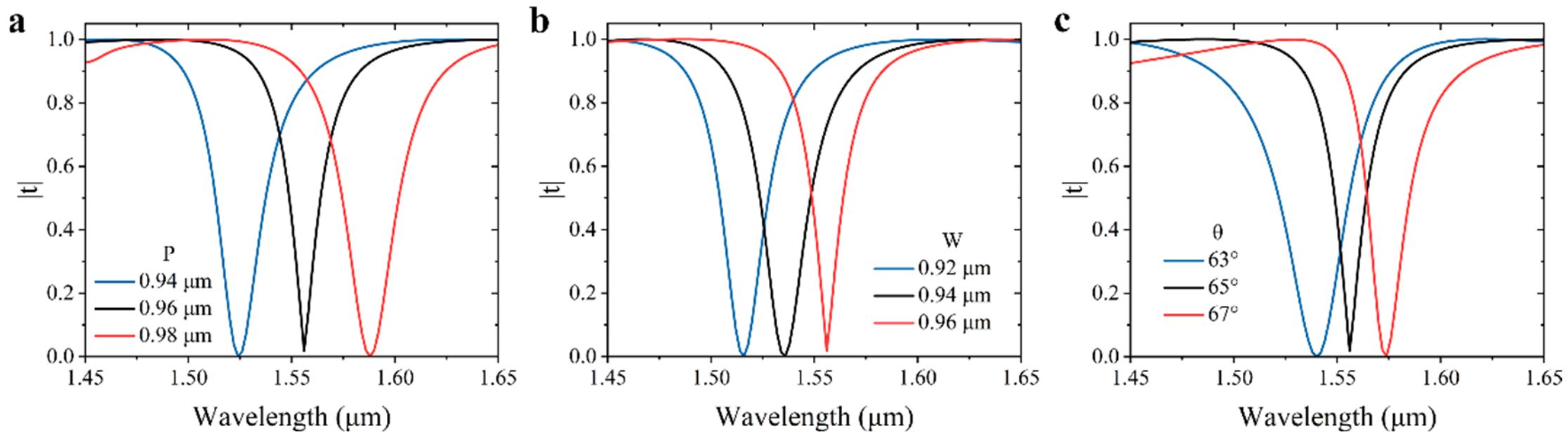

Figure S2. Relationship between metasurface transmission spectra and structure parameters. a) Transmission spectra of the metasurface with varying period P, under the condition L = P = W and θ = 65°. b) Transmission spectra with varying width W, under fixed L = P = 0.96 μm and θ = 65°. c) Transmission spectra with varying tilt angle θ, under fixed L = P = W = 0.96 μm.

Section S3: External voltage configurations for TFLN metasurface performing distinct mathematical operations

The TFLN metasurface designed in this work for mathematical operations consists of 80 identical structure units. Leveraging the electro-tunable properties of the TFLN metasurface structure and the dependence of its transmission coefficients on applied external voltages, distinct sets of external voltages can be applied to individual structure units to enable diverse mathematical functionalities. Specifically, when applying the voltage configurations listed in **Table S1** to each structure unit, the metasurface performs first-order differentiation. When applying the voltage configurations in **Table S2**, it achieves second-order differentiation. When applying the voltage configurations in **Table S3**, it executes integration. Consequently, simply reconfiguring the applied voltages allows dynamic switching of the TFLN metasurface between different mathematical operations.

Table S1. The applied voltage values for each structure unit in the first-order differentiation TFLN metasurface (*y* represents the coordinate value of the structure unit's center position, and U_{bias} denotes the external voltage applied to the TFLN structure at the corresponding position).

y(μm)	U_{bias}(V)	*y*(μm)	U_{bias}(V)	*y*(μm)	U_{bias}(V)	*y*(μm)	U_{bias}(V)
-37.92	-37.33	-18.72	-18.89	0.48	-0.9	19.68	17.16
-36.96	-36.39	-17.76	-17.98	1.44	0	20.64	18.07
-36	-35.45	-16.8	-17.08	2.4	0.89	21.6	18.99
-35.04	-34.51	-15.84	-16.18	3.36	1.79	22.56	19.91
-34.08	-33.58	-14.88	-15.27	4.32	2.69	23.52	20.83
-33.12	-32.65	-13.92	-14.37	5.28	3.59	24.48	21.75
-32.16	-31.72	-12.96	-13.47	6.24	4.49	25.44	22.67
-31.2	-30.79	-12	-12.57	7.2	5.39	26.4	23.59
-30.24	-29.86	-11.04	-11.67	8.16	6.29	27.36	24.52
-29.28	-28.94	-10.08	-10.77	9.12	7.19	28.32	25.45
-28.32	-28.02	-9.12	-9.87	10.08	8.09	29.28	26.38
-27.36	-27.1	-8.16	-8.98	11.04	8.99	30.24	27.31
-26.4	-26.18	-7.2	-8.08	12	9.89	31.2	28.25
-25.44	-25.27	-6.24	-7.18	12.96	10.8	32.16	29.19
-24.48	-24.35	-5.28	-6.28	13.92	11.7	33.12	30.12
-23.52	-23.44	-4.32	-5.38	14.88	12.61	34.08	31.07
-22.56	-22.53	-3.36	-4.49	15.84	13.52	35.04	32.01
-21.6	-21.61	-2.4	-3.59	16.8	14.43	36	32.96
-20.64	-20.71	-1.44	-2.69	17.76	15.34	36.96	33.91
-19.68	-19.8	-0.48	-1.8	18.72	16.25	37.92	34.87

Table S2. The applied voltage values for each structure unit in the second-order differentiation TFLN metasurface.

y(μm)	U_{bias}(V)	y(μm)	U_{bias}(V)	y(μm)	U_{bias}(V)	y(μm)	U_{bias}(V)
-37.92	34.87	-18.72	7.3	0.48	-1.2	19.68	8.21
-36.96	32.98	-17.76	6.43	1.44	-1.2	20.64	9.17
-36	31.16	-16.8	5.61	2.4	-1.2	21.6	10.18
-35.04	29.4	-15.84	4.84	3.36	-1.07	22.56	11.23
-34.08	27.69	-14.88	4.11	4.32	-0.89	23.52	12.33
-33.12	26.04	-13.92	3.43	5.28	-0.66	24.48	13.48
-32.16	24.44	-12.96	2.79	6.24	-0.39	25.44	14.68
-31.2	22.89	-12	2.2	7.2	-0.07	26.4	15.92
-30.24	21.4	-11.04	1.65	8.16	0.29	27.36	17.22
-29.28	19.95	-10.08	1.15	9.12	0.7	28.32	18.56
-28.32	18.56	-9.12	0.7	10.08	1.15	29.28	19.95
-27.36	17.22	-8.16	0.29	11.04	1.65	30.24	21.4
-26.4	15.92	-7.2	-0.07	12	2.2	31.2	22.89
-25.44	14.68	-6.24	-0.39	12.96	2.79	32.16	24.44
-24.48	13.48	-5.28	-0.66	13.92	3.43	33.12	26.04
-23.52	12.33	-4.32	-0.89	14.88	4.11	34.08	27.69
-22.56	11.23	-3.36	-1.07	15.84	4.84	35.04	29.4
-21.6	10.18	-2.4	-1.2	16.8	5.61	36	31.16
-20.64	9.17	-1.44	-1.2	17.76	6.43	36.96	32.98
-19.68	8.21	-0.48	-1.2	18.72	7.3	37.92	34.87

Table S3. The applied voltage values for each structure unit in the integration TFLN metasurface.

y(μm)	U_{bias}(V)	y(μm)	U_{bias}(V)	y(μm)	U_{bias}(V)	y(μm)	U_{bias}(V)
-37.92	-4.49	-18.72	-7.71	0.48	34.87	19.68	4.71
-36.96	-4.57	-17.76	-8.05	1.44	34.87	20.64	4.42
-36	-4.66	-16.8	-8.44	2.4	34.87	21.6	4.17
-35.04	-4.75	-15.84	-8.87	3.36	34.87	22.56	3.93
-34.08	-4.84	-14.88	-9.35	4.32	26.59	23.52	3.72
-33.12	-4.94	-13.92	-9.91	5.28	21.41	24.48	3.52
-32.16	-5.05	-12.96	-10.54	6.24	17.86	25.44	3.33
-31.2	-5.17	-12	-11.28	7.2	15.27	26.4	3.16
-30.24	-5.28	-11.04	-12.14	8.16	13.3	27.36	3
-29.28	-5.41	-10.08	-13.17	9.12	11.75	28.32	2.86
-28.32	-5.55	-9.12	-14.42	10.08	10.5	29.28	2.72
-27.36	-5.7	-8.16	-15.96	11.04	9.46	30.24	2.59
-26.4	-5.86	-7.2	-17.92	12	8.59	31.2	2.47
-25.44	-6.03	-6.24	-20.49	12.96	7.85	32.16	2.35
-24.48	-6.21	-5.28	-24.02	13.92	7.22	33.12	2.25
-23.52	-6.41	-4.32	-29.15	14.88	6.66	34.08	2.15
-22.56	-6.63	-3.36	-37.33	15.84	6.18	35.04	2.05
-21.6	-6.86	-2.4	-37.33	16.8	5.75	36	1.96
-20.64	-7.12	-1.44	-37.33	17.76	5.36	36.96	1.87
-19.68	-7.4	-0.48	-37.33	18.72	5.02	37.92	1.79

Section S4: The electrostatic field and refractive index distributions for the second-order differentiation TFLN metasurface

After applying external voltages, electrostatic field coupling occurs between structure units, leading to an inhomogeneous distribution of the electrostatic field and, consequently, an inhomogeneous refractive index of TFLN. Taking the second-order differentiation TFLN

metasurface as an example, **Figure S3**a shows the electrostatic field distribution within the metasurface structure after applying external voltages. It can be observed that, due to the coupling effect, the electrostatic field distribution in the TFLN structure is inhomogeneous, resulting in an inhomogeneous refractive index distribution, as shown in Figure S3b. However, simulation results demonstrate that this inhomogeneous refractive index distribution in the TFLN structure does not disrupt the analog computing function of the metasurface, particularly its first- and second-order differentiation capabilities.

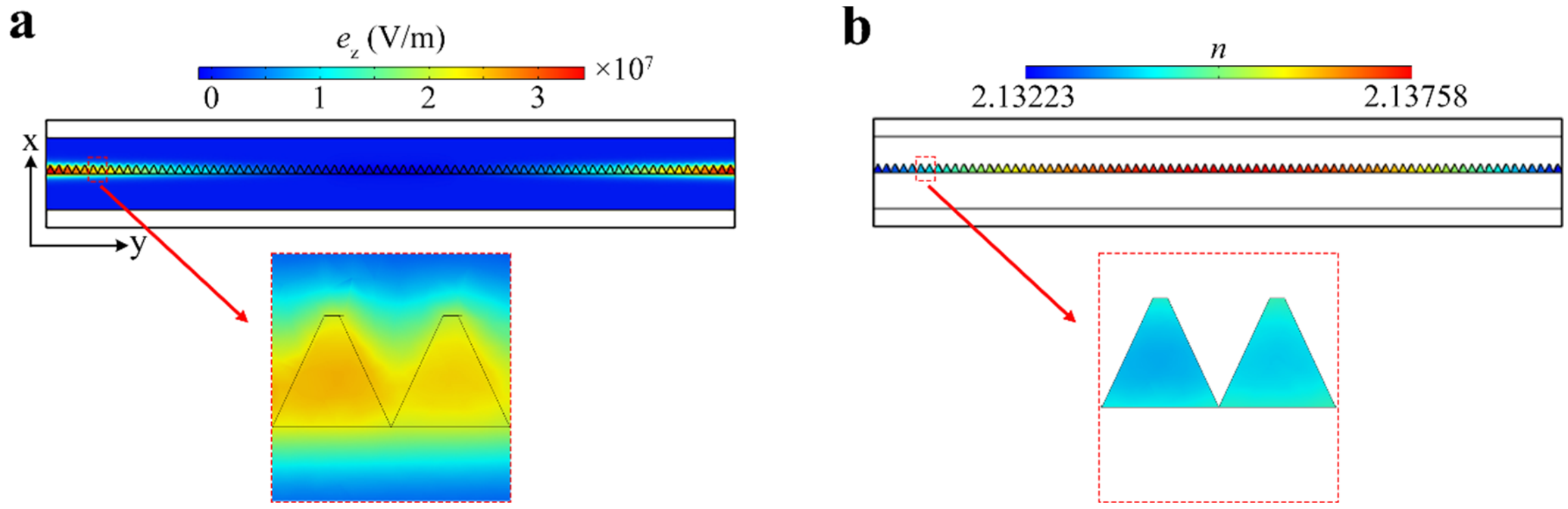

Figure S3. The electrostatic field and refractive index distributions for the second-order differentiation TFLN metasurface. a) The electrostatic field e_z distribution and b) the refractive index distribution of the TFLN structures for the second-order differentiation TFLN metasurface after applying external voltages. The insets within the red dashed boxes show enlarged views of the electrostatic field and refractive index distributions, respectively.

Section S5: Simulations performed using only the substrate without the TFLN structures

To further demonstrate the mathematical operation capabilities of the TFLN metasurface, we remove the TFLN structures from the metasurface while retaining the substrate. The electric field intensity $|E_z|^2$ distributions of the input rectangular signals with different widths are shown in **Figure S4**a–c corresponding to signal widths $2w_2$ of 40, 10, and 6 µm, respectively. Figure S4d–f display the electric field intensity distributions in the *y*–*z* plane at the output port. For enhanced visualization, the electric field intensity distributions in Figure S4a–f are generated by repeating 20 times along the *z*-axis. The output waveforms are approximately maintained as rectangular signals. However, due to the refractive index mismatch between the input-side air medium and substrate material, slight discrepancies exist between the output and input waveforms. Figure S4g–i show the line profiles along the *y*-axis of electric field intensities from Figure S4d–f, providing further evidence to support these conclusions.

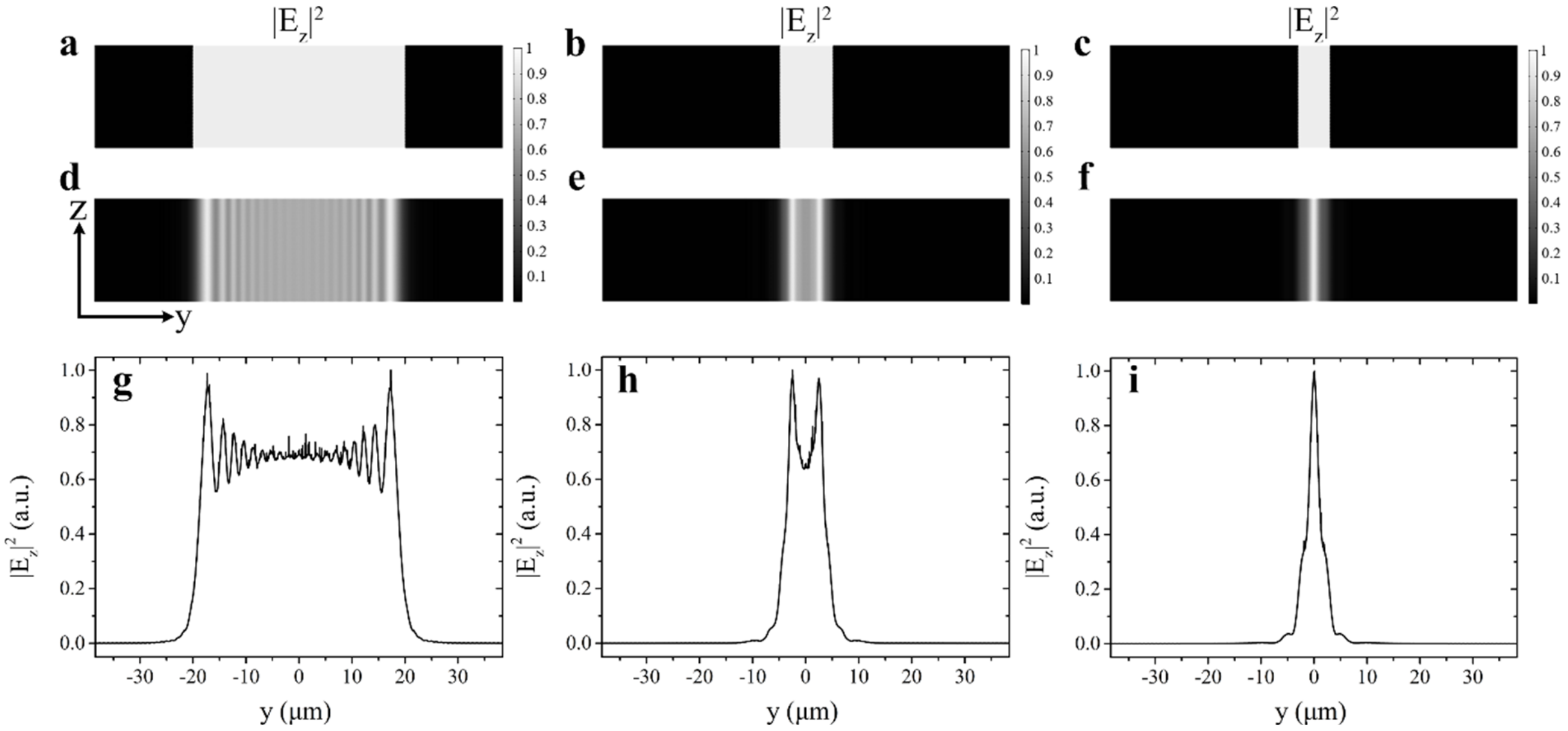

Figure S4. Simulations performed using only the substrate without the TFLN structures. a–c) Electric field intensity $|E_z|^2$ distributions of input rectangular signals with widths $2w_2$ of 40 μm, 10 μm, and 6 μm, respectively. d–f) Transmitted electric field intensity $|E_z|^2$ distributions at the output port after passing through the substrate. g–i) Line profiles along the *y*-axis of transmitted electric field intensity $|E_z|^2$ distributions corresponding to (d)–(f).

Section S6: Edge detection performance of the first-order differentiation TFLN metasurface

To verify its edge detection performance, rectangular signals are input into the first-order differentiation TFLN metasurface. **Figure S5**a–c show the electric field intensity $|E_z|^2$ distributions of the input rectangular signals with widths $2w_2$ of 40 μm, 20 μm, and 10 μm, respectively. Figure S5d–f display the electric field intensity distributions in the *y*–*z* plane at the output port. Clear bright lines emerge near the abrupt intensity transition regions of the rectangular signals, demonstrating that the edges of the signals are sharply resolved, or equivalently, the first-order differentiation TFLN metasurface successfully detects the signal edges. This conclusion is further validated by the line profiles of the transmitted electric field intensity distributions along the *y*-axis at the output port, as shown in Figure S5g–i. Comparison with Figure 5 in the main text indicates that the first-order and second-order differentiation operations of the proposed TFLN metasurface exhibit comparable edge detection performance.

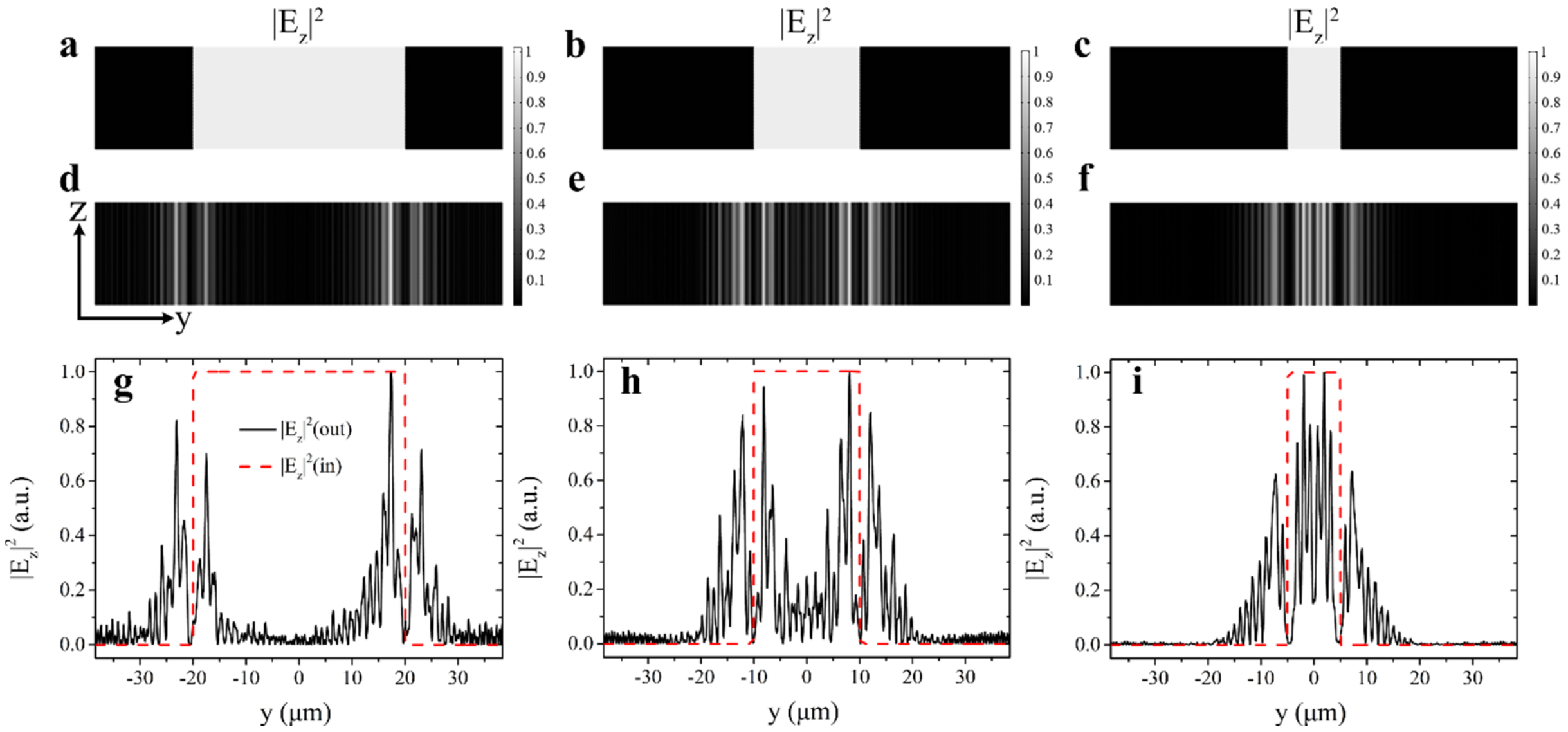

Figure S5. Edge detection performance of the first-order differentiation TFLN metasurface. a–c) Electric field intensity $|E_z|^2$ distributions of the input rectangular signals in the *y*–*z* plane, with signal widths $2w_2$ of 40 μm, 20 μm, and 10 μm, respectively. d–f) Transmitted electric field intensity $|E_z|^2$ distributions at the output port for the input rectangular signals in (a)–(c) after passing through the first-order differentiation TFLN metasurface. g–i) Line profiles (black solid lines) along the *y*-axis of transmitted electric field intensity $|E_z|^2$ distributions in (d)–(f). Red dashed lines represent the input rectangular signals.

Section S7: Tunability of the operating wavelength for the second-order differentiation TFLN metasurface

Due to the electrically tunable properties of the TFLN metasurface, the operating wavelength of its second-order differentiation function should theoretically be tunable. Through simulations, the relationship between the transmission coefficient t of the metasurface and the applied external voltage U_{bias} is obtained for *z*-polarized light at wavelengths of 1.554 μm and 1.556 μm incident vertically along the −*x*-axis, as shown in **Figure S6**a,b. By selecting $t_{max} = 0.2$ and $\angle t_{diff} = 90°$, appropriate external voltages are applied to individual structure units based on Figure S6a, ensuring that the transmission coefficient distribution of the TFLN metasurface satisfies Equation 3 and 4. For a *z*-polarized plane wave at 1.554 μm incident along the −*x*-axis, the simulated transmitted electric field E_z distribution in Figure S6c demonstrates the metasurface's second-order differentiation capability. As shown in Figure S6e,f, the line profiles along the *y*-axis at the output port of the E_z distribution shown in Figure S6c indicate that the simulation results (solid lines) align well with theoretical predictions (dashed lines), further confirming the metasurface's second-order differentiation functuion at 1.554 μm. Similarly, selecting $t_{max} = 0.2$ and $\angle t_{diff} = -90°$, and applying external voltages based on Figure S6b, the transmission coefficient distribution satisfies Equation 3 and 4 for a 1.556 μm *z*-polarized light. The simulated E_z distribution in Figure S6d validates the second-order differentiation operation at this wavelength. Corresponding line profiles along the *y*-axis in Figure S6g,h also show good agreement between simulations (solid lines) and theoretical predictions (dashed lines). These results demonstrate that by tuning the external voltages

applied to structure units, the operating wavelength of the second-order differentiation TFLN metasurface can be adjusted.

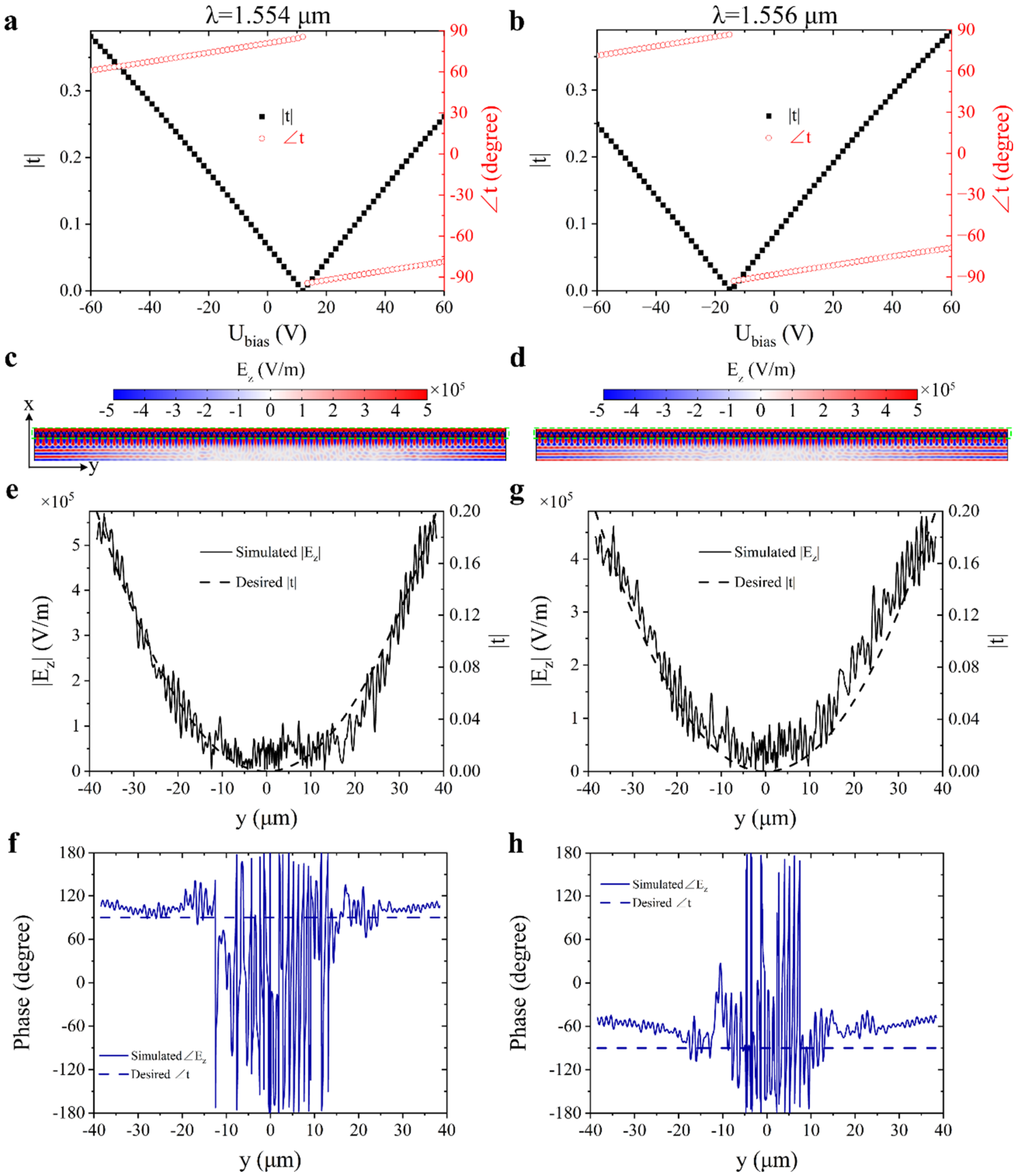

Figure S6. Tunability of the operating wavelength for the second-order differentiation TFLN metasurface. a,b) Relationships between the metasurface transmission coefficient t and the applied external voltage U_{bias} for *z*-polarized light at wavelengths of 1.554 μm and 1.556 μm, respectively, under incidence along the −*x*-axis. Black solid squares represent the amplitude of the transmission coefficient, while red open circles denote its phase. c,d) Transmitted electric field E_z distribution for *z*-polarized plane waves at 1.554 μm and 1.556 μm, respectively, incident along the −*x*-axis on the designed second-order differentiation TFLN metasurface. The TFLN metasurface structure is outlined by a green dashed box. e,f) Line profiles along the *y*-axis at the output port of the amplitude (black solid line) and phase (blue solid line) of the transmitted field E_z shown in (c). g,h) Line profiles along the *y*-axis at the output port of the amplitude (black solid line) and phase (blue solid line) of the transmitted field E_z shown in (d). Black and blue dashed lines represent the theoretically predicted amplitude and phase distributions of the transmission coefficient, respectively.

Section S8: The second-order differentiation of the designed TFLN metasurface when the incident wavelength deviates significantly from 1.555 μm

As indicated by the Figure 2, when the incident wavelength deviates significantly from 1.555 μm, the phase and amplitude modulation of the metasurface are no longer meet the requirements for analog computing. Consequently, the TFLN metasurface loses its original analog computing function under large wavelength deviations, as illustrated in Figure S7. Specifically, this figure shows the second-order differentiation of the designed TFLN metasurface ceases to function when illuminated by Gaussian beam $\exp[-(y/w_1)^2]$ (w_1 = 15 μm) at wavelengths of 1.4 μm and 1.7 μm.

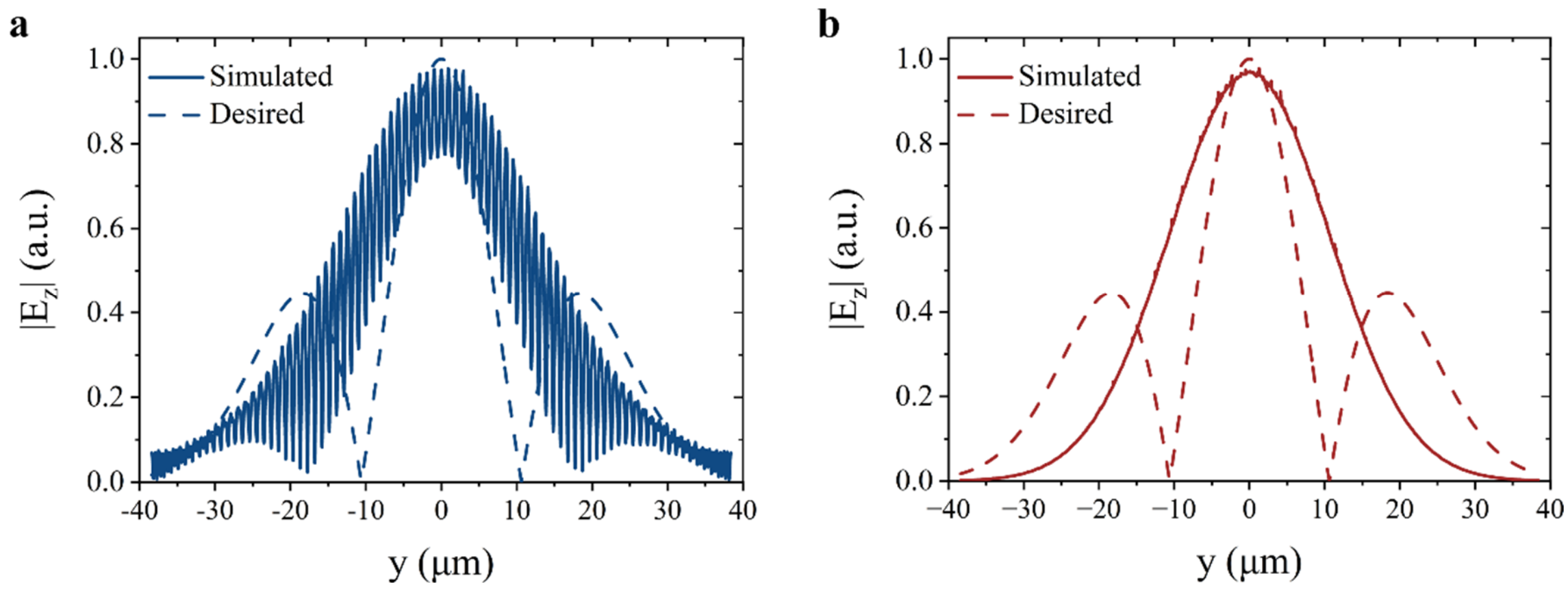

Figure S7. The second-order differentiation of the designed TFLN metasurface when the incident wavelength deviates significantly from 1.555 μm. a,b) Amplitude distributions of the output signals along the y-axis after passing through the second-order differentiation TFLN metasurface by inputting Gaussian beam signal $\exp[-(y/w_1)^2]$ (w_1 = 15 μm) at wavelengths of 1.4 μm and 1.7 μm, respectively. Solid lines represent simulation results, and dashed lines represent theoretical predictions.

References

1. Zelmon, D. E.; Small, D. L.; Jundt, D. *J. Opt. Soc. Am. B* **1997,** 14, (12), 3319–3322.
